# The evolutionary significance of fatigue

**DOI:** 10.3389/fphys.2013.00309

**Published:** 2013-10-31

**Authors:** Daniel A. Boullosa, Fábio Y. Nakamura

**Affiliations:** ^1^Departamento de Educação Física, Universidade Católica de BrasíliaTaguatinga, Brazil; ^2^Departamento de Educação Física, Universidade Estadual de LondrinaLondrina, Brazil

**Keywords:** perceived exertion, evolutionary biology, exercise physiology, human performance, Philosophy of Science

The concept of fatigue has inspired a wide body of research since the earliest studies in muscular and exercise physiology (Noakes, 2012). During the last decades, intense debates (Amann and Secher, 2010) have been conducted among sport and exercise physiologists in an attempt to clarify the mechanisms that regulate fatigue. It is interesting to note that various models have been proposed for its understanding along with the vast number of studies generated in this area (Abbiss and Laursen, 2005; Amann and Dempsey, 2008; Millet, 2011). Given this important lack of scientific consensus, it is therefore pertinent to clarify if we are thinking about the complex phenomenon of fatigue in the right way. From a historical perspective, the whole picture of fatigue research would evoke the old Indian tale of the blind men and the elephant. Briefly, this parable described several blind men touching an elephant to figure out what it looked like with a subsequent discussion on the nature of the elephant from their own perspectives. Every man touched only a single part of the elephant's body, leading each of them to guess differently about the object's features. Similarly, fatigue researchers have strongly defended different theories without assuming that they could only be aware of a limited piece of the whole fatigue phenomenon. Thus, the absence of agreement between fatigue specialists could have its origin with the technological dependence of fatigue research. That is, the epistemological approximation to fatigue has been more inductive than deductive as laboratory facilities with restricted findings (e.g., maximum oxygen uptake; VO_2_max) dictated the elaboration of further explanatory theories (e.g., Anaerobic/Cardiovascular Model). In this regard, some caution should be considered with the new technological advances in brain and muscle imaging as these could be performed under the same erroneous process. This consideration is very important given that new technologies are also capable of possessing important methodological limitations under exercise conditions that may limit our understanding of fatigue. Although it seems that we will see a new era in fatigue research, the risk of elaborating on biased knowledge because of technology limitations should not be ignored.

A new revolution in this area could start if the scientific process was more deductive. For example, a recent article has presented an evolutionary explanation for the greater effectiveness of different training and nutritional strategies (Boullosa et al., 2013). This line of reasoning has been inspired by previous works from evolutionary medicine based on the assumption that nothing in biology makes sense except in the light of evolution (Dobzhansky, 1973). That is, all biological phenomena are the consequences of millions of years of evolution through the action of natural selection (Lieberman, 2012). Therefore, as a biological phenomenon, fatigue should not be excluded from the influence of natural selection. The questions that arise include: how did fatigue evolve across evolutionary processes and what its evolutionary significance is? Consequently, it could be hypothesized that the protective role that fatigue exerts, developed to avoid a catastrophic failure of the organism. This assumption is one of the key points of the Central Governor Model (CGM) (Noakes, 2012). However, individuals are able to go beyond their body limits, despite fatigue symptoms, finally achieving the catastrophic failure (i.e., death) (Noakes, 2007). This apparent paradox could be overcome with the consideration of the *selfish gene hypothesis* (Dawkins, 1976). This hypothesis states that active replicators (i.e., genes), which are programmed to make copies of themselves, modulate the behavior of their carrier organism. The evolutionary significance of fatigue could be understood when assuming that its role on exercise performance (i.e., organism behavior) should be linked to the genetic success of the carrier organism (e.g., athlete). This assumption is critical and has important implications for designing appropriate studies in which the athlete is highly motivated during exercise under real conditions to better analyze the influence of fatigue on performance. However, it is well known that a more ecological approach limits the applicability of sophisticated and technological instruments. Moreover, it should be pointed out that true maximal efforts during exhaustive exercises are more suitable under real than under laboratory conditions as inferred from recorded maximum heart rate values in both conditions (Semin et al., 2008). Furthermore, an incremental test until exhaustion, which is a common protocol in fatigue evaluations, may have no equivalent in the evolutionary process of our species. These considerations raise important concerns regarding the validity of the common protocols in fatigue evaluation. This problem could be overcome in future studies with sufficient ecological validity while allowing the recording of valid data with new imaging technologies that should be interpreted at a translational level of physiology (Heck et al., 2011).

There are several findings that could reinforce the guiding role of this evolutionary approach in future fatigue research. For instance, it has been proposed that our ancestors predominantly performed physical activities at low intensities, mainly below lactate threshold (LT) (Boullosa et al., 2013). This concept supports the validity of *polarized training* that is widely utilized by modern endurance athletes and is characterized by keeping ’easy training easy, and hard training hard' (Muñoz et al., 2013). Within this concept, the main factor regulating the level of physical activity of our ancestors is proposed to be perception of effort (Smirmaul, 2012). Perception of effort increases in response to increased motor commands and cognitive effort related to maintenance of muscle contractions (Nakamura et al., 2008; Marcora et al., 2009). Locomotor muscle fatigue also increases perception of effort and reduces exercise tolerance (Marcora et al., 2008). As increased perception of effort is often accompanied by muscle contraction-induced discomfort and pain, it can inhibit engagement in purposeless behaviors which could undesirably lead to an increase in energy expenditure at a time when periods of food shortage were common. In other words, increased perception of effort during inefficient physical activities would be a favored phenotypic trait that evolved under scarce caloric sources, as energy efficiency is thought to be an important evolutionary pressure (Niven and Laughlin, 2008). It has to be pointed out that this view does not indicate that perception of effort has an anticipatory or template role that regulates exercise duration by means of subconscious control of motor commands (Swart et al., 2012). There is evidence supporting the more biologically plausible model that exercise intolerance is a cognitive event related to task disengagement due to attainment of maximal levels of perception of effort in highly motivated people (Marcora and Staiano, 2010). Subsequently, it is reasonable to imply that the modern athlete chooses to engage in activities below the LT most of the time as rating of perceived exertion (RPE) is maintained at tolerable and relatively comfortable values at this exercise level in an efficient manner in preparation for any following intense sessions. It is costly from a perceptual perspective to keep going for long periods of time on a daily basis above LT (Seiler and Tønnessen, 2009). Therefore, fatigue is a relevant issue that does change perception of effort and subsequent behavior in accordance with natural selection. This evidence could help to better understand the role of evolutionary concepts incorporated within this new era of fatigue research. More specifically, it would be pertinent to examine peripheral and central adaptations of athletes enrolled in long-lasting training regimes that consider this phylogenetic template (Boullosa et al., 2013). Greater adaptations and more efficient responses during maximal and submaximal exercises may become evident with the use of new imaging techniques in athletes when training within this concept.
